# Assessing coronavirus disease 2019 (COVID-19) transmission to healthcare personnel: The global ACT-HCP case-control study

**DOI:** 10.1017/ice.2020.455

**Published:** 2020-09-09

**Authors:** Robert J. Lentz, Henri Colt, Heidi Chen, Rosa Cordovilla, Spasoje Popevic, Sarabon Tahura, Piero Candoli, Sara Tomassetti, Gerard J. Meachery, Brandon P. Cohen, Bryan D. Harris, Thomas R. Talbot, Fabien Maldonado

**Affiliations:** 1 Division of Allergy, Pulmonary and Critical Care, Vanderbilt University Medical Center, Nashville, Tennessee, United States; 2 Departments of Thoracic Surgery, Vanderbilt University School of Medicine, Nashville, Tennessee, United States; 3 Department of Veterans’ Affairs Medical Center, Nashville, Tennessee, United States; 4 Division of Pulmonary Diseases and Critical Care Medicine (Emeritus), University of California, Irvine, California, United States; 5 Department of Biostatistics, Vanderbilt University Medical Center, Nashville, Tennessee, United States; 6 Department of Pulmonology, Interventional Pulmonary Unit, Salamanca University Hospital, Salamanca, Spain; 7 Department of Pulmonology, Interventional Pulmonology Unit, Clinical Center of Serbia, Belgrade, Serbia; 8 Department of Pediatric Respiratory Medicine, Bangladesh Institute of Child Health, Dhaka Shishu Hospital, Dhaka, Bangladesh; 9 Pulmonology Unit, Azienda Ospedali Riuniti Marche Nord, Pesaro, Italy; 10 Department of Experimental and Clinical Medicine, Interventional Pulmonary Unit, Careggi University Hospital, Florence, Italy; 11 Department of Respiratory Medicine and Cardiothoracic Transplantation, Institute of Transplantation, Freeman Hospital and Royal Victoria Infirmary, Newcastle Upon Tyne NHS Foundation Trust, United Kingdom; 12 Newcastle University, Newcastle Upon Tyne, United Kingdom; 13 HCA Healthcare, Ocala Health System, Ocala, Florida, United States; 14 Division of Infectious Diseases and Department of Infection Prevention, Vanderbilt University Medical Center, Nashville, Tennessee, United States

## Abstract

**Objective:**

To characterize associations between exposures within and outside the medical workplace with healthcare personnel (HCP) SARS-CoV-2 infection, including the effect of various forms of respiratory protection.

**Design:**

Case–control study.

**Setting:**

We collected data from international participants via an online survey.

**Participants:**

In total, 1,130 HCP (244 cases with laboratory-confirmed COVID-19, and 886 controls healthy throughout the pandemic) from 67 countries not meeting prespecified exclusion (ie, healthy but not working, missing workplace exposure data, COVID symptoms without lab confirmation) were included in this study.

**Methods:**

Respondents were queried regarding workplace exposures, respiratory protection, and extra-occupational activities. Odds ratios for HCP infection were calculated using multivariable logistic regression and sensitivity analyses controlling for confounders and known biases.

**Results:**

HCP infection was associated with non–aerosol-generating contact with COVID-19 patients (adjusted OR, 1.4; 95% CI, 1.04–1.9; *P* = .03) and extra-occupational exposures including gatherings of ≥10 people, patronizing restaurants or bars, and public transportation (adjusted OR range, 3.1–16.2). Respirator use during aerosol-generating procedures (AGPs) was associated with lower odds of HCP infection (adjusted OR, 0.4; 95% CI, 0.2–0.8, *P* = .005), as was exposure to intensive care and dedicated COVID units, negative pressure rooms, and personal protective equipment (PPE) observers (adjusted OR range, 0.4–0.7).

**Conclusions:**

COVID-19 transmission to HCP was associated with medical exposures currently considered lower-risk and multiple extra-occupational exposures, and exposures associated with proper use of appropriate PPE were protective. Closer scrutiny of infection control measures surrounding healthcare activities and medical settings considered lower risk, and continued awareness of the risks of public congregation, may reduce the incidence of HCP infection.

Understanding the epidemiology of coronavirus disease 2019 (COVID-19) including risk factors associated with COVID-19 in healthcare personnel (HCP) is critical because a substantial outbreak among HCP could dramatically disrupt patient care and threaten public health. Early reports have suggested that 19%–29% of COVID-19 cases involve HCP and that HCP are more likely to have an occupational-related infection than other professions.^1–3^ These findings align with data from the 2002–2003 severe acute respiratory syndrome (SARS) epidemic, in which HCP accounted for 21% of all global cases, most of which were believed to be nosocomial, and similar concerns have arisen amid the COVID-19 pandemic.^4,5^ Unlike SARS, however, COVID-19 achieved widespread community transmission around the world in early 2020, likely related to factors such as a propensity for asymptomatic and presymptomatic spread, placing HCP at risk for infection outside the workplace as well.^6–13^

In this study, we assessed the degree to which exposures within and outside the medical workplace may be associated with HCP COVID-19, and we investigated the association of different forms of respiratory protection on the odds of acquiring HCP infection. Explicit knowledge of exposures that place HCP at greater risk and what protective equipment reduces risk is of paramount importance to protect HCP. We hypothesized that healthcare activities capable of producing infectious aerosols would increase the odds of HCP infection while more protective respiratory PPE would reduce this risk.

## Methods

### Study design and participants

This international case-control study used an online survey (Supplement A online) to query HCP during a 2-week period between April 20, 2020, and May 5, 2020. HCP were defined as individuals working in healthcare delivery settings. All data collected were anonymous, and there was no direct investigator-to-respondent contact. The survey was conducted in REDCap.^14,15^ Invitations to participate (Supplement B online) were distributed by investigators from the United States, Spain, Italy, Serbia, and Bangladesh using HCP-oriented social media groups in WhatsApp, Facebook, Telegram, Reddit, and LinkedIn, as well as e-mail. After survey completion, respondents were encouraged to recruit local colleagues to maximize the likelihood of drawing cases and controls from the same population. Participants were not recruited based on any studied exposures. The study protocol was approved as exempt research by the Vanderbilt University Medical Center Institutional Review Board (VUMC IRB no. 200677).

### Variables and exposures

Respondents were required to confirm their status as HCP, then they were asked whether they (1) had been diagnosed with laboratory-confirmed COVID-19 (“cases”), (2) had experienced an illness suspicious for COVID-19 after January 1, 2020 that was not laboratory-confirmed (“possible cases”), or (3) had remained healthy while continuing to work (ie, “controls”). Laboratory-confirmed COVID-19 was defined as report of a polymerase chain reaction (PCR) test detecting severe acute respiratory coronavirus virus 2 (SARS-CoV-2). Cases and possible cases were asked the date of symptom onset and instructed to report their exposures during the 14 days prior to symptom onset. Controls were asked to complete the survey with respect to the 14 days prior to survey completion. A 14-day exposure window was chosen to correspond with the incubation period of SARS-CoV-2.^16^

The survey collected demographic data followed by questions about exposures to types of patients, healthcare settings, activities outside the workplace, and institutional policies regarding the use of PPE. Respondents exposed to laboratory-confirmed COVID-19 patients or persons under investigation for COVID-19 (COVID PUIs; defined as patients placed in precautionary isolation per local policy) were asked about specific exposures and respiratory protection used during the care of such patients. Intubation, extubation, open respiratory suctioning, bronchoscopy, nebulizer use, noninvasive positive pressure ventilation (NIPPV), tracheotomy, and cardiopulmonary resuscitation were considered aerosol-generating procedures (AGPs). Disposable N95, FFP2, and FFP3 respirators (new or reused), powered air-purifying respirators (PAPRs), and reusable elastomeric respirators were considered respirator-level protection. “Prolonged contact” with patients was defined as 45 minutes or longer.

### Statistical methods

Statistical analysis followed a prespecified analysis plan. “Possible cases” and HCP healthy throughout the pandemic who had not worked in the 14 days prior to survey completion were excluded, as were surveys missing any demographic or workplace exposure data.

Descriptive statistics included mean and standard deviation for age, and percentage and frequencies for categorical variables. Between group comparisons were conducted with Wilcoxon rank sum and Pearson χ^2^ tests for continuous and categorical variables, respectively. Odds ratios with 95% confidence intervals with respect to HCP COVID-19 were calculated for all exposures.

A multivariable logistic regression analysis was performed including prespecified confounders age, gender, smoking status, presence of a baseline comorbidity, healthcare role, and world region. Exposures involving COVID patients were additionally analyzed in a stratified manner according to the level of respiratory protection most frequently utilized during these exposures.

A prespecified sensitivity analysis was performed to detect temporal bias with respect to the date of case illness onset; we anticipated an incomplete overlap between case and control exposure time windows. Cases were grouped into the following cohorts and compared to controls: an “early sensitivity cohort” (symptom onset before April 1, 2020), “late sensitivity cohort” (onset on or after April 1, 2020), and “contemporaneous sensitivity cohort” (onset on or after April 20, 2020, the first day of survey data entry, therefore reporting over the same time period as controls).

## Results

**Fig. 1.**
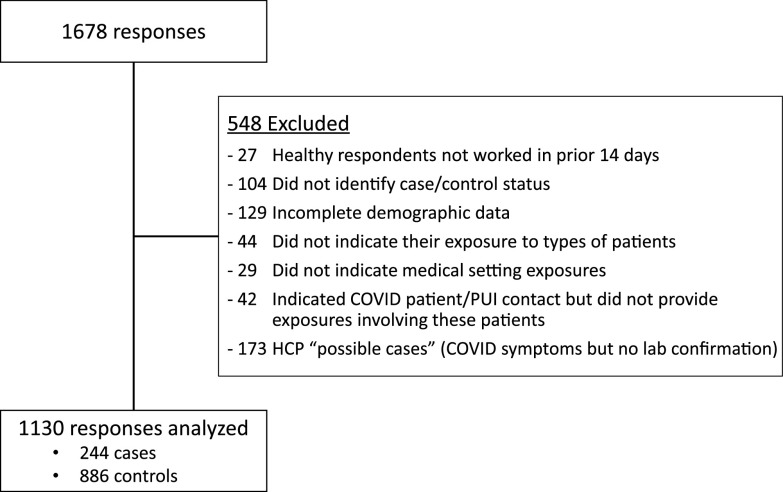
Study flow.

In total, 1,678 responses were received from 67 countries and 41 US states during the 2-week study period. Of these, 548 surveys were excluded from analysis based on prespecified eligibility criteria, including 173 “possible cases” (Fig. 1). Statistical analysis was performed on the remaining 1,130 records, including 244 cases and 886 controls, of which 147 had nonexclusionary missing data.

Cases and controls were similar demographically, except for a higher proportion of cases among nurses (41%) than clinicians (20%) or respiratory therapists (6%; *P* < .001) (Table 1). Just over half of the respondents were from Europe. Overall, the mean age of respondents was 42 years (range, 19–73); 62% were women; and 74% were physicians or midlevel providers. Approximately half of cases sought formal medical attention, and 23% required hospital admission (Appendix Table 1 online).

**Table 1.** Demographics of Cases and Controls

Characteristic	Cases (n=244), No. (%)	Controls (n=886), No. (%)
Age, mean y (±SD)	41 (±10)	43 (±11)
Sex, female	161 (66)	544 (61)
**Clinical role**		
Clinician^a^	165 (68)	668 (75)
Nurse	59 (24)	86 (10)
Respiratory therapist	5 (2)	81 (9)
Other^b^	15 (6)	51 (6)
**Any existing medical condition**	68 (28)	199 (22)
Asthma	23 (9)	60 (7)
Hypertension	26 (11)	87 (10)
Diabetes mellitus	8 (3)	26 (3)
Current or former smoker	51 (21)	208 (24)
**Region**		
Europe	144 (59)	465 (52)
North America	47 (19)	210 (24)
Asia	40 (16)	138 (16)
Other	13 (5)	73 (8)
**Institutional Setting**		
Major academic hospital	87 (36)	327 (37)
Major public hospital	64 (26)	241 (27)
Local public hospital	30 (12)	89 (10)
Major private hospital	28 (12)	78 (9)
Other^c^	35 (14)	151 (17)

Note. SD, standard deviation.

a Physicians, midlevel providers such as nurse practitioners, or other analogous roles.

b Includes administrative support staff, environmental services (custodial and janitorial), medical student or other clinical trainee, medical technician, emergency medical technician, medical laboratory staff, medical therapists, hospital transport staff.

c Includes community and local academic hospital, community and local private hospital, Veterans’ Affairs facility, military hospital, outpatient clinic, procedure center.

### Exposures to people in the healthcare workplace

Exposure to laboratory-confirmed COVID-19 patients was associated with HCP COVID-19 (adjusted OR, 1.4; 95% CI, 1.04–1.9; *P* = .046). Non-AGP exposure to COVID-19 patients (n = 634), but not participation in AGPs (n = 321), was associated with HCP infection, which persisted in multivariate and temporal sensitivity analyses (Table 2). Cases and controls did not differ in the number of COVID-19 patients cared for (mean, 19 vs 22; mean difference, 2.8; 95% CI, −2.7 to 8.2; *P* = .50). Workplace contact with an ill HCP colleague was associated with HCP infection (adjusted OR, 4.4; 95% CI, 3.2 to 6.0; *P* < .001).

**Table 2.** Odds Ratios for Healthcare Personnel (HCP) Infection Associated With Exposures to People in the Healthcare Setting

Variable	Unadjusted	*P* Value	Adjusted	*P* Value	Contemporaneous Sensitivity Cohort	*P* Value
COVID-19 patient	1.4 (1.1–1.9)	.02	1.4 (1.0–1.9)	.046	2.4 (1.2–4.5)	.01
AGP (n=321)	0.9 (0.6–1.2)	.39	0.9 0.6–1.2)	.44	1.0 (0.5–2.2)	.97
Non-AGP (n=634)	1.5 (1.1–2.0)	.009	1.4 (1.04–1.9)	.03	2.2 (1.2–4.1)	.01
COVID PUI	0.9 (0.7–1.3)	.64	0.9 (0.6–1.2)	.36	2.5 (1.0–6.1)	.04
AGP (n=288)	0.8 (0.5–1.1)	.09	0.8 (0.5–1.1)	.12	0.7 (0.3–1.4)	.31
Non-AGP (n=759)	1.0 (0.8–1.4)	.86	1.0 (0.7–1.3)	.76	2.1 (0.98–4.5)	.06
Non-COVID patient	0.7 (0.5–1.0)	.06	0.8 (0.6–1.1)	.22	0.5 (0.2–0.9)	.02
Sick colleague (n=459)^a^	4.9 (3.6–6.6)	<.001	4.4 (3.2–6.0)	<.001	4.8 (2.5–9.2)	<.01

Note. AGP, aerosol-generating procedure; PUI, person under investigation for COVID-19.

a COVID symptoms or diagnosed with confirmed COVID-19.

### Respiratory protection and specific exposures to COVID-19 patients

Respirators were used by 94% of respondents during AGPs and 72% during non-AGPs. Respirator use in both AGPs and non-AGPs was associated with being a control (adjusted OR, 0.4; 95% CI, 0.2–0.8; *P* = .005), whereas the use of medical masks in both was associated with HCP infection (adjusted OR, 7.4; 95% CI, 2.8–20.0; *P* < .001) (Table 3). Among those with only non-AGP contact, use of a medical mask was associated with HCP infection in the univariate but not adjusted or temporal sensitivity analyses. The reuse of disposable respirators during AGPs (vs use of new disposable respirators) was not significantly associated with HCP infection (n = 254; adjusted OR, 1.6; 95% CI, 0.7–3.5; *P* = .29).

**Table 3.** Respiratory Protection Utilized Most Frequently During Contact With COVID-19 Patients

				All Cases, Unadjusted^a^		All Cases, Adjusted		Early Sensitivity Cohort^b^		Late Sensitivity Cohort^c^		Contemporaneous Cohort^d^	
During AGPs		During Non-AGPs	Total	OR	*P* Value	OR	*P* Value	OR	*P* Value	OR	*P* Value	OR	*P* Value
Respirator	+	Respirator	242	0.4 (0.2–0.8)	.003	0.4 (0.2–0.8)	.005	0.2 (0.06–0.5)	<.001	0.7 (0.3–1.5)	.35	0.5 (0.1–2.4)	.42
Respirator	+	Medical mask	55	1.0 (0.5–2.1)	.99	1.0 (0.4–2.3)	.94	1.1 (0.3–3.9)	.84	0.8 (0.3–2.1)	.68	0.6 (0.06–5.2)	.62
Medical mask	+	Medical mask	18	7.4 (2.8–20.0)	<.001	9.1 (2.8–29.9)	<.001	38.3 (7.7–189.9)	<.001	3.9 (1.0–14.6)	.04	4.7 (0.4–53.2)	.21
(No exposure)	+	Respirator	208	1.1 (0.7–1.6)	.67	1.2 (0.8–1.9)	.43	0.8 (0.4–1.6)	.48	1.3 (0.8–2.0	.36	1.6 (0.7–3.4)	.23
(No exposure)	+	Medical mask	99	1.7 (1.1–2.7)	.02	1.6 (0.9–2.6)	.09	1.9 (0.9–3.9)	.10	1.2 (0.6–2.1)	.65	1.1 (0.5–2.9)	.80

a Median exposure windows Apr 14–28, 2020 (controls, n=886) and Mar 20–Apr 3, 2020 (all cases, n = 244).

b Median case exposure window Mar 3–17, 2020 (n=101).

c Median case exposure window Apr 3–17, 2020 (n=141).

d Median case exposure window Apr 10–24, 2020 (n=54).

Prolonged contact with COVID-19 patients was associated with HCP infection in both univariate and temporal sensitivity analyses (Appendix Table 2 online). The odds of HCP infection were greater in those reporting prolonged continuous COVID-19 patient contact without a respirator (adjusted OR, 2.3; 95% CI, 1.1–4.9; *P* = .04) versus those who wore respirators in this context (adjusted OR, 0.8, 95% CI, 0.5–1.5; *P* = .60). Caring for COVID-19 patients in negative pressure rooms was associated with being a control (OR range, 0.4–0.7 across analyses). Most AGPs demonstrated ORs <1.0 though many did not meet statistical significance (Appendix Table 2 online).

### Exposures to specific healthcare settings

Working in ICUs, dedicated COVID ICUs, or dedicated COVID wards was associated with significantly lower odds of HCP COVID-19 (adjusted OR range, 0.5–0.7; 95% CI, 0.3–0.96; *P* < .05) than exposure to regular hospital wards (adjusted OR, 1.4; 95% CI, 1.0–1.9; *P* = .05) (Appendix Table 3 online). Skilled nursing or long-term care facility exposure also associated with HCP infection (adjusted OR, 2.9; 95% CI, 1.3–6.4; *P* = .007).

### Exposures outside the healthcare workplace

Exposure to ill household members, gatherings of ≥10 people, patronizing restaurants or bars, and public transportation was associated with HCP infection (Table 4). Adjusted odds ratios ranging from 4.6 to 16.2 for the latter 3 exposures decreased over time in the temporal sensitivity analysis (contemporaneous cohort OR range, 3.1–4.7), though remained significantly associated with HCP infection (all *P* < .05).

**Table 4.** Odds Ratios Associated with Extra-occupational and Local Institutional Policy Exposures

Variable	All Cases, Unadjusted	*P* Value	All Cases, Adjusted	*P* Value	Early Sensitivity Cohort	*P* Value	Late Sensitivity Cohort	*P* Value	Contemporaneous Sensitivity Cohort	*P* Value
**Exposures outside work**										
Person with known COVID-19	1.5 (0.95–2.5)	.08	1.5 (0.9–2.5)	.10	2.1 (1.2–4.0)	.02	1.0 (0.5–2.1)	.99	0.7 (0.2–2.9)	.58
Person with COVID symptoms	1.2 (0.8–1.8)	.42	1.1 (0.7–1.7)	.75	1.9 (1.1–3.2)	.02	0.7 (0.4–1.2)	.20	0.2 (0.02–1.2)	.07
Household member, known COVID-19	4.4 (1.9–10.3)	<.001	3.8 (1.5–9.3)	.004	4.1 (1.3–13.9)	.02	3.6 (1.3–10.4)	.02	4.2 (0.8–22.0)	.09
Household member, COVID symptoms	3.0 (1.6–5.8)	<.001	3.1 (1.5–6.3)	.002	6.3 (2.8–14.2)	<.001	1.6 (0.5–4.6)	.42	1.4 (0.2–11.1)	.76
Gathering of ≥10 people	4.6 (3.1–7.0)	<.001	4.6 (3.0–7.1)	<.001	8.6 (5.0–14.9)	<.001	2.4 (1.4–4.4)	.002	3.4 (1.5–7.8)	.003
Patronized restaurant or bar^a^	15.8 (8.6–29.3)	<.001	16.2 (8.6–30.5)	<.001	45.3 (21.7–94.8)	<.001	3.6 (1.5–8.8)	.005	4.7 (1.5–15.1)	.008
In-person retail shopping	0.9 (0.6–1.2)	.35	0.9 (0.6–1.2)	.36	1.0 (0.6–1.6)	.93	0.8 (0.5–1.2)	.21	1.2 (0.6–2.1)	.66
Used public transportation	5.4 (3.5–8.2)	<.001	4.4 (2.8–6.9)	<.001	7.9 (4.3–14.4)	<.001	2.7 (1.5–4.7)	<.001	3.1 (1.3–7.0)	.009
**Institutional policies**										
Respirator for AGP	0.4 (0.3–0.7)	<.001	0.4 (0.3–0.7)	<.001	0.3 (0.2–0.6)	<.001	0.7 (0.4–1.2)	.19	0.8 (0.3–2.1)	.63
Respirator for non-AGP	0.6 (0.4–0.8)	<.001	0.6 (0.5–0.9)	.008	0.6 (0.4–0.97)	.04	0.7 (0.4–1.0)	.06	1.0 (0.5–2.0)	.93
Re-use of disposable respirators	1.1 (0.8–1.5)	.60	1.1 (0.8–1.5)	.74	1.0 (0.6–1.5)	.82	1.1 (0.7–1.7)	.60	1.5 (0.8–3.0)	.22
No PPE doffing between patients^b^	0.9 (0.6–1.2)	.33	0.9 (0.7–1.2)	.50	0.8 (0.5–1.3)	.30	1.1 (0.7–1.6)	.73	0.7 (0.3–1.4)	.28
In-person PPE training	1.3 (0.9–1.7)	.13	1.1 (0.8–1.6)	.44	2.1 (1.3–3.4)	.001	0.7 (0.4–0.99)	.05	0.8 (0.4–1.5)	.41
PPE observers always utilized^c^	0.4 (0.3–0.7)	<.001	0.4 (0.3–0.7)	.001	0.1 (0.04–0.5)	.002	0.7 (0.4–1.2)	.19	0.4 (0.1–1.5)	.16

Note. AGP, aerosol-generating procedure; PPE, personal protective equipment.

a Includes only dine-in or drink-in, not take-away.

b On dedicated COVID units.

c Compared to PPE observers never utilized.

### Exposures to local institutional policies

Working at facilities with policies recommending respirator use during AGPs (adjusted OR, 0.4; 95% CI, 0.3–0.7; *P* < .001) and non-AGP contact (adjusted OR, 0.6; 95% CI, 0.5–0.9; *P* = .008) were associated with being a control, while HCP at institutions with policies advocating extended use or reuse of disposable respirators did not associate with being a case or control (Table 4). HCP reporting that they were always observed donning and doffing PPE by dedicated observers were more likely to be controls than those who reported PPE observers were never available (adjusted OR, 0.4; 95% CI, 0.3–0.7; *P* = .001).

## Discussion

Results from this international case-control study of >1,100 HCP from >60 countries characterizing important exposures associated with HCP COVID-19 may have immediate implications for infection control policies within and outside the healthcare setting. First, our results indicate that nosocomial transmission to HCP was more likely during routine contact with COVID-19 patients than during AGPs. Second, significantly lower likelihood of HCP infection was associated with working in ICUs and COVID units, respirator use (in some contexts), and dedicated PPE observers, which reinforces the protective value of being familiar with and using appropriate PPE. Third, multiple exposures outside the healthcare setting were strongly associated with HCP infection, suggesting that transmission of COVID-19 in the community remains a critical and underappreciated contributor to HCP infection.

Within the healthcare setting, AGPs have been implicated as major risk factors for nosocomial transmission of respiratory viral infections such as SARS-CoV-1.^17^ Accordingly, attention has focused on practices believed to protect against transmission during these procedures, including use of respirators, negative pressure ventilation, and procedural techniques hypothesized to reduce aerosolization.^18–21^ Perhaps related to such attention, our results indicate that participation in AGPs was not associated with HCP infection. Protective associations noted for respirator use during COVID-19 AGPs and work in ICUs and dedicated COVID units (the latter corroborated by a recent brief report utilizing PCR data^22^) suggest that appropriate PPE and familiarity with its use and the risks involved in COVID-19 patient care are likely highly protective, which is further bolstered by the protective association with dedicated PPE observers. These data suggest that providers can perform COVID-19 patient care, including AGPs, confidently with appropriate PPE including respirators, training, and supervision.

Routine non-AGP contact with COVID-19 patients, however, was associated with HCP infection in this study. Optimal respiratory protection during non-AGP contact with COVID-19 patients remains uncertain. The World Health Organization recommends medical masks in this context, whereas the US Centers for Disease Control and Prevention advises that respirators are preferred.^23,24^ A meta-analysis of 4 pre–COVID-19 randomized trials involving 4,531 subjects suggested that respirators and medical masks offer similar protection from a variety of respiratory viral infections, though the certainty of this finding was considered low.^25^ Another meta-analysis of 44 observational studies involving protection from epidemic β-coronaviruses indicated that respirators may be more protective than face masks, but it only included a single COVID-19–specific study related to this topic, which retrospectively compared respirator use to no mask use.^26,27^

Our study represents the only COVID-19–specific data comparing respirators to other forms of respirator protection. Non-AGP medical mask use was not consistently associated with HCP COVID-19, but HCP infection was more likely with prolonged continuous contact (≥45 m) while not wearing a respirator (adjusted OR, 2.3 vs 0.8 when respirator used). These results are consistent with a more nuanced view of respiratory viral transmission in which the distinction between large droplet and small-particle aerosol is better described as a spectrum than dichotomous mechanisms of infection.^28–31^ Respiratory viral transmission requires sufficient contamination of respiratory mucus membranes by virus-laden large droplets or deposition of virus-containing aerosol small particles in the lower respiratory tract. Inhaled dose would be expected to increase with higher concentrations of aerosolized viral particles and longer duration of exposure but decreases with effective filtration of inhaled air. This finding aligns with observed patterns of HCP infection in our study: (1) AGPs increase the concentration of aerosolized virus and are associated with HCP infection unless highly effective filtration (a respirator) is utilized. (2) Negative pressure ventilation reduces aerosolized viral burden and is associated with protection from infection. And (3) prolonged non-AGP contact without a respirator might occasionally allow a sufficient inhaled dose and is associated with modestly increased odds ratio for HCP COVID-19. Our results suggest that medical masks are likely adequate during most non-AGP contact with COVID-19 patients, but respirators might be considered if very prolonged close contact is anticipated.

Some HCP who responded to our survey certainly acquired SARS-CoV-2 outside the workplace. Several odds ratios corresponding to extra-occupational exposures decreased in magnitude in the temporal sensitivity analysis due to the reduced frequency of these exposures over time as stay-at-home orders and business closures escalated. The odds ratios associated with gatherings of ≥10 people, dining in a restaurant or patronizing a bar, and using public transportation remained high, even in the contemporaneous sensitivity cohort, where they exceeded the odds ratios for all occupational exposures except AGP exposure without respirator protection. These results suggest that extra-occupational exposures remain highly pertinent to HCP safety and indicate a continued risk in scenarios involving public congregation.

Finally, close contact with afflicted individuals in settings without the same PPE expectations as those during patient care were associated with HCP infection, notably exposure to sick HCP colleagues and household members. Protective behaviors, including social distancing outside the patient–provider relationship and not working while ill, remain pertinent to HCP, particularly in light of a recent report noting that 65% of HCP with COVID-19 worked while symptomatic.^32^

The strengths of this study include its large international sample of HCP, befitting of a pandemic with global consequences, which ensures good generalizability across a broad range of healthcare systems. Controls were matched as closely as possible to cases by design in which cases were asked to recruit local controls. Most analyzed responses (87%) contained complete data, and 242 of 375 responses excluded by missing data criteria were nearly empty responses, lacking case-control or demographic information, which was ascertained at the start of the survey. Data on exposures and respiratory protections utilized were collected in a specific and detailed manner, and statistical analysis proceeded according to prespecified analysis plan including multivariate and temporal sensitivity analyses. This study also exemplifies a design in which medically oriented social media facilitated rapid and far-reaching HCP recruitment across a broad range of geographic origin and healthcare roles.

Although a case-control design is appropriate for a study intended to rapidly collect data from an international sample involving relatively rare individual events, this design has limitations. Confirming respondent SARS-CoV-2 status was not practical from a timing, logistic, or economic perspective given the diverse nature of this sample, so we relied on self-report of laboratory-confirmed COVID-19. A “possible case” group, which was then excluded from the primary analysis, was used to avoid these respondents from erroneously indicating that they were laboratory-confirmed cases, and the large sample size limits the influence of any uncommon erroneous self-reports. Without serologic testing, the issue of asymptomatic infection remains, and some crossover of occult infection into the control group almost certainly occurred, which would be expected to dampen the odds ratios. Recall bias is possible, although this is not dissimilar from other key studies used to assess HCP risk of SARS acquisition.^33^ Controls were asked to report their most proximate exposures to limit recall bias. This design resulted in incomplete overlap in case and control exposure windows, addressed by a prespecified temporal sensitivity analysis. Geographic bias caused by differing levels of community illness and risk of exposure across different regions was addressed by including region in the multivariate logistic regression, though more local variations may have had some impact. We intended to capture comprehensive data relating to HCP infection risk, but the possibility of unmeasured confounders is always present. Additionally, some exposures noted to be protective from infection, such as use of negative pressure rooms, might also be reflective of more advanced or affluent healthcare systems, which may also reduce HCP infection in other ways (eg, access to more or higher quality PPE, for example). Finally, respondents self-selected into study participation, so selection or collider bias is possible. This factor is among the reasons that case-control studies detect associations but do not imply causation, and we have been careful to discuss our results in a manner befitting the certainty level appropriate to this study design.

Results from this international case-control study highlight several occupational and extra-occupational exposures associated with symptomatic COVID-19 among HCP. Attention should now shift to currently less-scrutinized “lower risk” activities and hospital settings. Implementation of PPE observer programs may help address occupational risk, while HCP should remain vigilant for potential exposures outside of work which were more associated with infection than most healthcare exposures in this study. These results have immediate implications for healthcare and public policy.

## Supplementary material

For supplementary material accompanying this paper visit http://dx.doi.org/10.1017/ice.2020.455. click here to view supplementary material
