# Supplementary material for: Assessing coronavirus disease 2019 (COVID-19) transmission to healthcare personnel: The global ACT-HCP case-control study
Source: Infect Control Hosp Epidemiol. 2020 Sep 9:1–7. doi: 10.1017/ice.2020.455 (PMC7542323; doi:10.1017/ice.2020.455)
Supplement: Supplementary file 1 [file icesup.zip › S0899823X20004559sup001.docx]

**Appendix**

Assessing COVID-19 Transmission to Healthcare Personnel: the Global ACT-HCP Case-Control Study

Contents

[Supplement A: Survey tool used for data collection 2](#_Toc42608891)

[Supplement B: Survey invitation 2](#_Toc42608892)

[Appendix Table 1. Clinical features of HCP COVID-19 infections 3](#_Toc42608893)

[Appendix Table 2. Odds ratios associated with specific exposures to COVID-19 patients 4](#_Toc42608894)

[Appendix Table 3. Odds ratios associated with exposure to healthcare settings 7](#_Toc42608895)

### Supplement A: Survey tool used for data collection

See separate file. Note: this survey was administered electronically, which allowed use of “branching logic” in which some questions or comment boxes would only appear if pertinent based on prior responses entered into the survey. For example, the second item in the survey, a comment box starting with “You have indicated that you are not a healthcare worker. This survey is for healthcare workers only” only appeared if the respondent selected that they were not a healthcare worker in the first question.

### Supplement B: Survey invitation

See separate file. Disseminated electronically via HCP-specific groups/channels in WhatsApp, Facebook, Telegram, Reddit, and LinkedIn as well as via email.

### Appendix Table 1. Clinical features of HCP COVID-19 infections

|  | **Cases** (n=244) |
| --- | --- |
| **Symptom** |  |
| Fever | 58% (142) |
| Cough | 57% (138) |
| Fatigue | 57% (140) |
| Headache | 48% (116) |
| Myalgias and/or arthralgias | 41% (100) |
| Anosmia | 40% (98) |
| Nasal / sinus congestion | 34% (83) |
| Sore throat | 30% (74) |
| Shortness of breath | 28% (67) |
| Diarrhea | 28% (68) |
| Chills | 21% (51) |
| Nausea and/or vomiting | 14% (35) |
| Other symptoms | 14% (33) |
| Abdominal pain | 8% (20) |
| **Severity of illness** |  |
| Minimally symptomatic | 12% (26) |
| Mild (average cold) | 25% (54) |
| Moderate (average flu) | 33% (71) |
| Moderately-severe (worse than average flu) | 28% (61) |
| Severe or critical illness | 2% (5) |
| **Disposition** |  |
| Self-managed at home | 55% (121) |
| Outpatient visit | 12% (26) |
| ER / ED / A&E visit | 10% (21) |
| Admission to a regular ward/floor | 16% (34) |
| Admission to a step-down unit | 6% (13) |
| Admission to intensive care unit | 1% (2) |

Percents (n).

### Appendix Table 2. Odds ratios associated with specific exposures to COVID-19 patients

| **Exposure** | **All Cases - Unadjusted** | **P** | **All Cases - Adjusted** | **P** | **Contemporaneous Sensitivity Cohort** | **p** |
| --- | --- | --- | --- | --- | --- | --- |
| **Inside a negative-pressure room with a patient** | 0.635 (0.427 to 0.945) | 0.0252 | 0.684 (0.445 to 1.051) | 0.0832 | 0.4 (0.16 to 1.0) | 0.05 |
| Respirator | 0.519 (0.309 to 0.869) | 0.0127 | 0.512 (0.28 to 0.936) | 0.0296 |  |  |
| Non-respirator | 0.499 (0.212 to 1.177) | 0.1125 | 0.638 (0.227 to 1.792) | 0.3935 |  |  |
| **Inside a non-negative-pressure room with a patient** | 1.185 (0.891 to 1.577) | 0.2438 | 1.132 (0.836 to 1.531) | 0.4230 | 1.581 (0.912 to 2.741) | 0.1029 |
| Respirator | 0.777 (0.472 to 1.278) | 0.3203 | 0.812 (0.477 to 1.384) | 0.4444 |  |  |
| Non-respirator | 1.204 (0.64 to 2.266) | 0.5646 | 1.300 (0.631 to 2.68) | 0.4770 |  |  |
| **Within 3 feet of a patient** | 1.134 (0.854 to 1.506) | 0.3856 | 1.071 (0.792 to 1.447) | 0.6565 | 1.088 (0.628 to 1.885) | 0.7640 |
| Respirator | 0.667 (0.391 to 1.138) | 0.1377 | 0.600 (0.336 to 1.072) | 0.0847 |  |  |
| Non-respirator | 0.853 (0.418 to 1.74) | 0.6620 | 0.815 (0.341 to 1.948) | 0.6453 |  |  |
| **With patient continuously for ≥45 min** | 1.532 (1.098 to 2.138) | 0.0120 | 1.331 (0.93 to 1.905) | 0.1174 | 1.001 (0.493 to 2.03) | 0.9988 |
| Respirator | 1.049 (0.653 to 1.685) | 0.8432 | 0.859 0.504 to 1.466 | 0.5780 |  |  |
| Non-respirator* | 2.349 (1.196 to 4.616) | 0.0132 | 2.259 (1.052 to 4.851) | 0.0366 |  |  |
| **With patient(s) cumulatively >1h during day/shift** | 1.407 (1.022 to 1.937) | 0.0361 | 1.185 (0.84 to 1.671) | 0.3340 | 2.057 (1.158 to 3.655) | 0.0139 |
| Respirator | 1.235 (0.781 to 1.951) | 0.3665 | 1.048 (0.627 to 1.751) | 0.8585 |  |  |
| Non-respirator | 1.319 (0.681 to 2.558) | 0.4120 | 1.003 (0.48 to 2.094) | 0.9940 |  |  |
| **Physical contact with patient** | 1.266 (0.952 to 1.685) | 0.1055 | 1.164 (0.857 to 1.581) | 0.3309 | 0.965 (0.55 to 1.696) | 0.9025 |
| Respirator | 0.965 (0.587 to 1.587) | 0.8884 | 0.819 (0.471 to 1.423) | 0.4784 |  |  |
| Non-respirator | 1.204 (0.628 to 2.309) | 0.5763 | 1.353 (0.654 to 2.797) | 0.4151 |  |  |
| **Physical contact with patient belongings** | 1.123 (0.805 to 1.567) | 0.4960 | 0.868 (0.606 to 1.241) | 0.4372 | 1.232 (0.657 to 2.311) | 0.5154 |
| Respirator | 0.736 (0.456 to 1.19) | 0.2110 | 0.525 (0.307 to 0.899) | 0.0188 |  |  |
| Non-respirator | 1.330 (0.701 to 2.523) | 0.3833 | 0.947 (0.453 to 1.983) | 0.8859 |  |  |
| **Physical contact with patient’s respiratory secretions** | 0.907 (0.61 to 1.348) | 0.6290 | 0.789 (0.517 to 1.203) | 0.2703 | 0.308 (0.095 to 1.0) | 0.050 |
| Respirator | 0.548 (0.312 to 0.96) | 0.0354 | 0.482 (0.262 to 0.885) | 0.0187 |  |  |
| Non-respirator | 1.663 (0.777 to 3.559) | 0.1901 | 1.254 (0.533 to 2.951) | 0.6039 |  |  |
| **Performed intubation** | 0.725 (0.383 to 1.372) | 0.3229 | 0.818 (0.424 to 1.579) | 0.5498 | 0.264 (0.036 to 1.946) | 0.1915 |
| Respirator | 1.092 (0.509 to 2.344) | 0.8205 | 0.999 (0.436 to 2.293) | 0.9989 |  |  |
| Non-respirator |  |  |  |  |  |  |
| **Intubation witness or assistant** | 0.741 (0.437 to 1.258) | 0.2665 | 0.769 (0.439 to 1.346) | 0.3573 | 0.547 (0.167 to 1.791) | 0.3188 |
| Respirator | 0.875 (0.446 to 1.717) | 0.6977 | 0.708 (0.33 to 1.518) | 0.3749 |  |  |
| Non-respirator |  |  |  |  |  |  |
| **Performed extubation** | 0.552 (0.246 to 1.24) | 0.1501 | 0.628 (0.272 to 1.448) | 0.2749 | 0.353 (0.048 to 2.608) | 0.3073 |
| Respirator | 0.631 (0.235 to 1.693) | 0.3603 | 0.644 (0.224 to 1.851) | 0.4141 |  |  |
| Non-respirator |  |  |  |  |  |  |
| **Extubation witness or assistant** | 1.291 (0.675 to 2.47) | 0.4396 | 1.034 (0.521 to 2.054) | 0.9237 | 1.350 (0.402 to 4.527) | 0.6271 |
| Respirator | 1.965 (0.912 to 4.235) | 0.0846 | 1.469 (0.622 to 3.467) | 0.3806 |  |  |
| Non-respirator |  |  |  |  |  |  |
| **Performed open suctioning** | 0.552 (0.302 to 1.009) | 0.053 | 0.477 (0.254 to 0.896) | 0.0214 | 0.377 (0.09 to 1.577) | 0.1815 |
| Respirator | 0.412 (0.177 to 0.959) | 0.0396 | 0.312 (0.121 to 0.804) | 0.0159 |  |  |
| Non-respirator |  |  |  |  |  |  |
| **Performed closed suctioning** | 0.887 (0.567 to 1.387) | 0.5992 | 0.879 (0.544 to 1.42) | 0.5969 | 0.134 (0.018 to 0.982) | 0.0480 |
| Respirator | 0.704 (0.395 to 1.256) | 0.2353 | 0.736 (0.391 to 1.387) | 0.3426 |  |  |
| Non-respirator | 1.028 (0.388 to 2.725) | 0.9558 | 1.083 (0.327 to 3.587) | 0.8960 |  |  |
| **Present during delivery of nebulized medication** | 1.114 (0.689 to 1.802) | 0.6587 | 1.108 (0.666 to 1.845) | 0.6927 | 0.193 (0.026 to 1.413) | 0.1052 |
| Respirator | 0.783 (0.387 to 1.584) | 0.4961 | 0.772 (0.36 to 1.657) | 0.5068 |  |  |
| Non-respirator |  |  |  |  |  |  |
| **Present during NIPPV use** | 0.713 (0.49 to 1.038) | 0.0775 | 0.688 (0.461 to 1.025) | 0.0661 | 0.455 (0.192 to 1.079) | 0.0738 |
| Respirator | 0.602 (0.323 to 1.123) | 0.1109 | 0.578 (0.278 to 1.203) | 0.1426 |  |  |
| Non-respirator |  |  |  |  |  |  |
| **Adjusted or applied patient’s NIPPV mask** | 0.602 (0.38 to 0.952) | 0.0301 | 0.539 (0.331 to 0.878) | 0.0130 | 0.441 (0.157 to 1.242) | 0.1211 |
| Respirator | 0.558 (0.297 to 1.047) | 0.0691 | 0.438 (0.206 to 0.935) | 0.0327 |  |  |
| Non-respirator |  |  |  |  |  |  |
| **Present during HFNC use** | 1.045 (0.744 to 1.468) | 0.8001 | 1.028 (0.714 to 1.479) | 0.8829 | 0.919 (0.465 to 1.815) | 0.8069 |
| Respirator | 1.004 (0.543 to 1.859) | 0.9890 | 1.033 (0.525 to 2.03) | 0.9261 |  |  |
| Non-respirator | 1.731 (0.858 to 3.491) | 0.1255 |  |  |  |  |
| **Adjusted or applied patient’s HFNC** | 0.977 (0.649 to 1.469) | 0.9094 | 0.865 (0.555 to 1.347) | 0.5205 | 0.898 (0.397 to 2.032) | 0.7968 |
| Respirator | 1.004 (0.543 to 1.859) | 0.9890 | 0.976 (0.481 to 1.982) | 0.9467 |  |  |
| Non-respirator | 1.648 (0.718 to 3.783) | 0.2386 |  |  |  |  |
| **Performed bronchoscopy** | 0.551 (0.295 to 1.031) | 0.0623 | 0.646 (0.338 to 1.234) | 0.1857 | 0.410 (0.098 to 1.716) | 0.2222 |
| Respirator | 0.590 (0.272 to 1.279) | 0.1812 | 0.780 (0.327 to 1.858) | 0.5744 |  |  |
| Non-respirator |  |  |  |  |  |  |
| **Bronchoscopy witness or assistant** | 0.699 (0.323 to 1.511) | 0.3620 | 0.632 (0.285 to 1.401) | 0.2590 | 0.389 (0.052 to 2.882) | 0.3554 |
| Respirator | 0.478 (0.163 to 1.406) | 0.1801 | 0.412 (0.133 to 1.279) | 0.1251 |  |  |
| Non-respirator |  |  |  |  |  |  |
| **Performed rigid bronchoscopy** | 0.256 (0.034 to 1.959) | 0.1895 | 0.329 (0.042 to 2.555) | 0.2879 |  |  |
| Respirator |  |  |  |  |  |  |
| Non-respirator |  |  |  |  |  |  |
| **Rigid bronchoscopy witness or assistant** | 1.090 (0.298 to 3.994) | 0.8960 | 0.813 (0.207 to 3.188) | 0.7667 |  |  |
| Respirator |  |  |  |  |  |  |
| Non-respirator |  |  |  |  |  |  |
| **Performed open tracheotomy** | 1.038 (0.214 to 5.028) | 0.9633 | 1.043 (0.196 to 5.549) | 0.9610 |  |  |
| Respirator |  |  |  |  |  |  |
| Non-respirator |  |  |  |  |  |  |
| **Open tracheotomy witness or assistant** | 0.990 (0.274 to 3.578) | 0.9880 | 0.878 (0.225 to 3.424) | 0.8508 |  |  |
| Respirator |  |  |  |  |  |  |
| Non-respirator |  |  |  |  |  |  |
| **Performed percutaneous tracheostomy** | 0.452 (0.056 to 3.629) | 0.4547 | 0.420 (0.051 to 3.448) | 0.4196 |  |  |
| Respirator |  |  |  |  |  |  |
| Non-respirator |  |  |  |  |  |  |
| **Percutaneous tracheostomy witness or assistant** | 0.775 (0.221 to 2.72) | 0.6911 | 0.896 (0.247 to 3.244) | 0.8672 |  |  |
| Respirator |  |  |  |  |  |  |
| Non-respirator |  |  |  |  |  |  |
| **Participated in CPR; no advanced airway at some point** | 1.319 (0.607 to 2.864) | 0.4841 | 1.233 (0.544 to 2.797) | 0.6158 | 0.650 (0.086 to 4.889) | 0.6754 |
| Respirator | 1.261 (0.45 to 3.536) | 0.6591 | 1.006 (0.325 to 3.118) | 0.9915 |  |  |
| Non-respirator |  |  |  |  |  |  |
| **Participated in CPR; advanced airway throughout** | 1.056 (0.496 to 2.25) | 0.8871 | 1.114 (0.507 to 2.45) | 0.7877 | 0.520 (0.07 to 3.886) | 0.5243 |
| Respirator | 1.326 (0.543 to 3.241) | 0.5353 | 1.196 (0.451 to 3.171) | 0.7188 |  |  |
| Non-respirator |  |  |  |  |  |  |
| **Recognized PPE breach during any COVID contact** | 1.026 (0.606 to 1.738) | 0.9236 | 0.981 (0.56 to 1.72) | 0.9468 | 0.612 (0.183 to 2.051) | 0.4263 |
| Respirator | 0.797 (0.398 to 1.597) | 0.5223 | 0.730 0.293 to 1.82 | 0.4997 |  |  |
| Non-respirator | 1.834 (0.703 to 4.783) | 0.2150 | 1.741 (0.61 to 4.967) | 0.3000 |  |  |

*These significant associations with HCP infection in adjusted and unadjusted analyses persisted even after the 18 respondents who wore medical masks during aerosol generating procedures were excluded (OR 2.4, 1.2 to 5.0, p=0.02 and adjusted OR 2.3, 1.02 to 5.4, p=0.04)

Cells with no data had too few cases for meaningful statistical analysis.

### Appendix Table 3. Odds ratios associated with exposure to healthcare settings

|  | **Unadjusted** | **p** | **Adjusted** | **p** | **Contemporary Sensitivity Analysis** | **p** |
| --- | --- | --- | --- | --- | --- | --- |
| ICU | 0.47 (0.34 to 0.66) | <0.001 | 0.574 (0.404 to 0.817) | 0.002 | 0.370 (0.171 to 0.802) | 0.0118 |
| Dedicated COVID-ICU | 0.44 (0.28 to 0.68) | <0.001 | 0.485 (0.308 to 0.765) | 0.0019 | 0.293 (0.088 to 0.979) | 0.0462 |
| Stepdown unit | 0.64 (0.45 to 0.89) | 0.008 | 0.760 (0.532 to 1.085) | 0.1308 | 0.237 (0.09 to 0.621) | 0.0034 |
| Regular Ward | 1.22 (0.91 to 1.62) | 0.18 | 1.360 (0.997 to 1.856) | 0.0524 | 1.339 (0.716 to 2.506) | 0.3612 |
| Dedicated COVID ward | 0.60 (0.42 to 0.84) | 0.003 | 0.672 (0.47 to 0.962) | 0.0296 | 0.517 (0.24 to 1.118) | 0.0936 |
| ER / A&E | 1.06 (0.77 to 1.47) | 0.7 | 1.146 (0.819 to 1.604) | 0.4258 | 1.045 (0.533 to 2.05) | 0.8983 |
| Out of hospital EMS | 1.04 (0.56 to 1.92) | 0.9 | 0.987 (0.528 to 1.845) | 0.9678 | 0.728 (0.168 to 3.161) | 0.6715 |
| Operating/procedure area | 0.66 (0.45 to 0.95) | 0.03 | 0.682 (0.463 to 1.003) | 0.0519 | 0.494 (0.202 to 1.207) | 0.1218 |
| Outpatient setting* | 1.14 (0.83 to 1.56) | 0.4351 | 1.320 (0.936 to 1.863) | 0.1136 | 0.994 (0.513 to 1.927) | 0.9869 |
| SNF or LTAC | 3.53 (1.68 to 7.43) | <0.001 | 2.931 (1.34 to 6.407) | 0.0071 | ‡ |  |
| Dialysis unit | 0.36 (0.08 to 1.54) | 0.17 | 0.431 (0.098 to 1.896) | 0.2657 | 0.550 (0.067 to 4.544) | 0.5792 |
| Home health | 0.96 (0.35 to 2.58) | 0.93 | 1.011 (0.364 to 2.81) | 0.9832 | 0.825 (0.098 to 6.977) | 0.8598 |
| Rehab facility | 0.78 (0.22 to 2.72) | 0.69 | 1.090 (0.302 to 3.931) | 0.8950 | ‡ |  |
| Medical laboratory | 0.69 (0.23 to 2.02) | 0.49 | 0.665 (0.217 to 2.04) | 0.4759 | 1.280 (0.248 to 6.61) | 0.7686 |
| Non-patient care area† | 0.40 (0.16 to 1.02) | 0.055 | 0.460 (0.178 to 1.187) | 0.1083 | 0.394 (0.051 to 3.018) | 0.3699 |
| Administrative area | 1.50 (0.68 to 3.31) | 0.31 | 1.647 (0.73 to 3.718) | 0.2295 | 1.798 (0.478 to 6.764) | 0.3852 |

*Clinic, testing area, or other ambulatory setting

†e.g. Radiology reading room

‡Too few (<15) respondents with this exposure
