# Supplementary material for: Assessing coronavirus disease 2019 (COVID-19) transmission to healthcare personnel: The global ACT-HCP case-control study
Source: Infect Control Hosp Epidemiol. 2020 Sep 9:1–7. doi: 10.1017/ice.2020.455 (PMC7542323; doi:10.1017/ice.2020.455)
Supplement: Supplementary file 1 [file icesup.zip › S0899823X20004559sup002.pdf]

ACT-HCP Study

Assessing COVID-19 Transmission to HealthCare Personnel (the ACT-HCP Study)Survey Purpose: Identify exposures associated with higher risk of SARS-CoV-2 transmission to healthcare workers.This survey was IRB-approved by Vanderbilt University Medical Center as exempt research.

This survey is anonymous.

We will NOT ask for your name, institution, or any information which might possibly identify you.

You may fill this survey out whether you have had COVID-19 or have remained healthy during this pandemic.

If you have had COVID-19, answer the questions regarding the 14-day time period before onset of your symptoms.If you have been healthy, answer questions with respect to your experiences during the last 14 days.Please forward this survey URL on to any colleagues you know who have been ill with COVID-19!

Questions: robert.j.lentz@vumc.org, henricolt@gmail.com, fabien.maldonado@vumc.org

Link to full study protocol.

Deadline for participation: Monday, May 4 (today!)

Inclusion and COVID-19 Infection Status

I work in a healthcare or medical setting (I am a healthcare worker)	◯ Yes<br>◯ No
You have indicated that you are not a healthcare worker. This survey is for healthcare workers only.<br><br>If you work a healthcare setting in any capacity (hospital, clinic, medical office, or laboratory), select "YES" for the question above.	
Have you been diagnosed with laboratory-confirmed COVID-19 at any point during the current pandemic?	◯ Yes<br>◯ No<br>◯ No, but I have experienced an illness I suspect was COVID-19
Were you tested for COVID-19?	◯ Yes, I tested negative<br>◯ Yes, the result is still pending<br>◯ No

Please consider bookmarking this survey URL and returning to fill out the survey once your COVID-19 test has returned. Laboratory-confirmation of COVID-19 will be important to know for the analysis of this study!

What symptoms did you experience?	☐ I was asymptomatic<br>☐ Fever<br>☐ Cough<br>☐ Shortness of breath<br>☐ Headache<br>☐ Sore throat<br>☐ Nasal / sinus congestion<br>☐ Myalgias and/or arthralgias<br>☐ Fatigue<br>☐ Nausea and/or vomiting<br>☐ Diarrhea<br>☐ Abdominal pain<br>☐ Chills<br>☐ Anosmia<br>☐ Other symptoms

You have indicated that you suffered from an illness you suspect was COVID but that you had no symptoms. Please double-check your answers above!

What kind of laboratory test returned positive to definitively establish your diagnosis?<br><br>The PCR test is the only widely-available test. It is usually run on a nasopharyngeal, nasal, or oropharyngeal swab. If you are not sure what kind of test you had, select "PCR."<br><br>(PCR = polymerase chain reaction)	◯ PCR<br>◯ Serology / antibody test<br>◯ Both PCR and serology
Rate your illness severity	◯ Minimally symptomatic<br>◯ Mild symptoms, akin to an average cold<br>◯ Moderate symptoms, akin to an average flu<br>◯ Moderately-severe symptoms, worse than the average flu<br>◯ Severe or critical illness
What is the maximal level of medical attention / care your illness required?	◯ Self-managed at home<br>◯ Outpatient visit (in-person to via telemedicine)<br>◯ ER / ED / A&E visit<br>◯ Admission to a regular ward/floor<br>◯ Admission to a step-down unit<br>◯ Admission to intensive care unit
Have you been to work at some point in the last 14 days?	◯ Yes<br>◯ No

You have indicated that you have not been to work in the last two weeks. The virus causing COVID-19 tends to have an incubation time within 2 weeks, and this is a study of healthcare-associated exposures. Please bookmark this URL and return to complete the survey within two weeks of working in a healthcare setting.

Today's Date	______________________________
Date of COVID-19 symptom onset<br><br>(approximate date is fine)	______________________________

Date of your positive COVID-19 PCR test?

(approximate date is fine)

____

Please complete the rest of this survey with respect to exposures and institutional policies in place during the 14 days PRIOR TO ONSET of your symptoms.

This 14 day time period is referred to as the "time period of interest" in the questions that follow.

Please complete the rest of this survey with respect to exposures and institutional policies in place during the 14 days PRIOR TO YOUR POSITIVE COVID-19 PCR test.

This 14 day time period is referred to as the "time period of interest" in the questions that follow.

You have indicated that you never had COVID-19 symptoms but have tested positive for COVID-19 by serologic testing. Because you never had symptoms, it is not possible to narrow down the window of when you might have been exposed to the virus. Please complete the rest of this survey with respect to exposures you experienced at work since January 2020 (ignore any reference to "14 day time period"). For the institutional policy questions, indicate the policies currently in place at your institution.

Please complete the rest of this survey with respect to exposures and institutional policies in place during the LAST 14 days.

This 14 day time period is referred to as the "time period of interest" in the questions that follow.

Geography/setting

World region

- ◯ Asia
- ◯ Africa
- ◯ Central America / Caribbean
- ◯ Europe
- ◯ Northern America
- ◯ South America
- ◯ Australia / Oceania

Country

- ◯ Afghanistan
- ◯ Albania
- ◯ Algeria
- ◯ American Samoa (USA)
- ◯ Andorra
- ◯ Angola
- ◯ Antigua and Barbuda
- ◯ Argentina
- ◯ Armenia
- ◯ Australia
- ◯ Austria
- ◯ Azerbaijan
- ◯ Bahamas
- ◯ Bahrain
- ◯ Bangladesh
- ◯ Barbados
- ◯ Belarus
- ◯ Belgium
- ◯ Belize
- ◯ Benin
- ◯ Bhutan
- ◯ Bolivia
- ◯ Bosnia and Herzegovina
- ◯ Botswana
- ◯ Brazil
- ◯ Brunei
- ◯ Bulgaria
- ◯ Burkina Faso
- ◯ Burundi
- ◯ Cabo Verde
- ◯ Cambodia
- ◯ Cameroon
- ◯ Canada
- ◯ Central African Republic
- ◯ Chad
- ◯ Chile
- ◯ Colombia
- ◯ Comoros
- ◯ Congo
- ◯ Costa Rica
- ◯ C??d'Ivoire
- ◯ Croatia
- ◯ Cuba
- ◯ Cyprus
- ◯ Czech Republic
- ◯ Denmark
- ◯ Djibouti
- ◯ Dominica
- ◯ Dominican Republic
- ◯ DR Congo
- ◯ Ecuador
- ◯ Egypt
- ◯ El Salvador
- ◯ Equatorial Guinea
- ◯ Eritrea
- ◯ Estonia
- ◯ Eswatini
- ◯ Ethiopia
- ◯ Fiji
- ◯ Finland
- ◯ France
- ◯ Gabon
- ◯ Gambia
- ◯ Georgia
- ◯ Germany
- ◯ Ghana
- ◯ Greece
- ◯ Greenland (Denmark)
- ◯ Grenada

◯ Guam (USA)
◯ Guatemala
◯ Guinea
◯ Guinea-Bissau
◯ Guyana
◯ Haiti
◯ Holy See
◯ Honduras
◯ Hong Kong (China)
◯ Hungary
◯ Iceland
◯ India
◯ Indonesia
◯ Iran
◯ Iraq
◯ Ireland
◯ Israel
◯ Italy
◯ Jamaica
◯ Japan
◯ Jordan
◯ Kazakhstan
◯ Kenya
◯ Kiribati
◯ Kuwait
◯ Kyrgyzstan
◯ Laos
◯ Latvia
◯ Lebanon
◯ Lesotho
◯ Liberia
◯ Libya
◯ Liechtenstein
◯ Lithuania
◯ Luxembourg
◯ Madagascar
◯ Malawi
◯ Malaysia
◯ Maldives
◯ Mali
◯ Malta
◯ Marshall Islands
◯ Mauritania
◯ Mauritius
◯ Mexico
◯ Micronesia
◯ Moldova
◯ Monaco
◯ Mongolia
◯ Montenegro
◯ Morocco
◯ Mozambique
◯ Myanmar
◯ Namibia
◯ Nauru
◯ Nepal
◯ Netherlands
◯ New Zealand
◯ Nicaragua
◯ Niger
◯ Nigeria
◯ North Korea
◯ North Macedonia
◯ Norway
◯ Oman
◯ Pakistan
◯ Palau
◯ Panama
◯ Papua New Guinea
◯ Paraguay
◯ Peru

◯ Philippines
◯ Poland
◯ Portugal
◯ Puerto Rico (USA)
◯ Qatar
◯ Romania
◯ Russia
◯ Rwanda
◯ Saint Kitts & Nevis
◯ Saint Lucia
◯ Samoa
◯ San Marino
◯ Sao Tome & Principe
◯ Saudi Arabia
◯ Senegal
◯ Serbia
◯ Seychelles
◯ Sierra Leone
◯ Singapore
◯ Slovakia
◯ Slovenia
◯ Solomon Islands
◯ Somalia
◯ South Africa
◯ South Korea
◯ South Sudan
◯ Spain
◯ Sri Lanka
◯ St. Vincent & Grenadines
◯ State of Palestine
◯ Sudan
◯ Suriname
◯ Sweden
◯ Switzerland
◯ Syria
◯ Taiwan
◯ Tajikistan
◯ Tanzania
◯ Thailand
◯ Timor-Leste
◯ Togo
◯ Tonga
◯ Trinidad and Tobago
◯ Tunisia
◯ Turkey
◯ Turkmenistan
◯ Tuvalu
◯ U.S. Virgin Islands (USA)
◯ Uganda
◯ Ukraine
◯ United Arab Emirates
◯ United Kingdom
◯ United States
◯ Uruguay
◯ Uzbekistan
◯ Vanuatu
◯ Venezuela
◯ Vietnam
◯ Yemen
◯ Zambia
◯ Zimbabwe

State	◯ Alabama<br>◯ Alaska<br>◯ Arizona<br>◯ Arkansas<br>◯ California<br>◯ Colorado<br>◯ Connecticut<br>◯ Delaware<br>◯ Florida<br>◯ Georgia<br>◯ Hawaii<br>◯ Idaho<br>◯ Illinois<br>◯ Indiana<br>◯ Iowa<br>◯ Kansas<br>◯ Kentucky<br>◯ Louisiana<br>◯ Maine<br>◯ Maryland<br>◯ Massachusetts<br>◯ Michigan<br>◯ Minnesota<br>◯ Mississippi<br>◯ Missouri<br>◯ Montana<br>◯ Nebraska<br>◯ Nevada<br>◯ New Hampshire<br>◯ New Jersey<br>◯ New Mexico<br>◯ New York<br>◯ North Carolina<br>◯ North Dakota<br>◯ Ohio<br>◯ Oklahoma<br>◯ Oregon<br>◯ Pennsylvania<br>◯ Rhode Island<br>◯ South Carolina<br>◯ South Dakota<br>◯ Tennessee<br>◯ Texas<br>◯ Utah<br>◯ Vermont<br>◯ Virginia<br>◯ Washington<br>◯ Washington, D.C.<br>◯ West Virginia<br>◯ Wisconsin<br>◯ Wyoming
What type of hospital do you work in?	◯ Major/referral hospital - Academic<br>◯ Major/referral hospital - Private<br>◯ Major/referral hospital - Public/Government<br>◯ Community/local - Academic<br>◯ Community/local - Private<br>◯ Community/local - Public/Government<br>◯ Veteran's Affairs<br>◯ Military Hospital<br>◯ Outpatient clinic or procedure center<br>◯ Other or not applicable

Your demographics

Age

____
(years)

Gender

- ◯ Male
- ◯ Female
- ◯ Non-binary or other

What is your job / role in the healthcare setting?

- ◯ Clinician (physician, mid-level provider, other similar)
- ◯ Nurse
- ◯ Respiratory Therapist
- ◯ Medical technician with patient contact (e.g. EKG tech, ultrasonographer)
- ◯ Medical technician without patient contact (e.g. telemetry monitor)
- ◯ Administrative support with patient contact
- ◯ Administrative support without patient contact
- ◯ Medical student or other clinical trainee
- ◯ Environmental services or janitorial
- ◯ Other

Other medical role:

____

Do you have any chronic medical conditions?

- ◯ Yes
- ◯ No

Comorbidities: select all that apply	☐ ---- RESPIRATORY ----<br>☐ Chronic obstructive pulmonary disease (COPD)<br>☐ Asthma<br>☐ Chronic hypoxemic respiratory failure (any etiology)<br>☐ Active tuberculosis infection<br>☐ ---- CARDIOVASCULAR ----<br>☐ Hypertension (HTN)<br>☐ Heart failure (including reduced or preserved ejection fraction)<br>☐ Coronary Artery Disease (CAD) or Peripheral Arterial Disease (PAD)<br>☐ Cardiac arrhythmia<br>☐ ---- ENDOCRINE ----<br>☐ Diabetes mellitus<br>☐ Obesity (body mass index >30)<br>☐ ---- MALIGNANCY ----<br>☐ Solid tumor malignancy (current and/or active)<br>☐ Leukemia or lymphoma (current and/or active)<br>☐ ---- NEUROLOGIC / COGNITIVE ----<br>☐ Dementia<br>☐ History of stroke / cerebrovascular accident (CVA)<br>☐ ---- HEPATIC ----<br>☐ Chronic liver disease with cirrhosis<br>☐ Chronic liver disease without cirrhosis<br>☐ ---- RENAL ----<br>☐ Chronic kidney disease<br>☐ End-stage renal disease on renal replacement therapy<br>☐ ---- RHEUMATIC / CONNECTIVE TISSUE DISEASE ----<br>☐ Any rheumatic / connective tissue disease<br>☐ ---- IMMUNOCOMPROMISE ----<br>☐ History of bone marrow / stem cell transplant<br>☐ History of solid organ transplant<br>☐ Human Immunodeficiency Virus (HIV) infection<br>☐ Immunocompromise related to drugs not for transplant<br>☐ Other immunocompromised state<br>☐ ---- OTHER CORMORBIDITIES ----<br>☐ Other notable comorbidity(ies)
Clarify "other" response; one comorbidity per line	__________________________________
Tobacco smoking status or smoking history	◯ Current smoker<br>◯ Former smoker<br>◯ Never smoker
Did you take any doses of hydroxychloroquine or chloroquine during the 14-day time interval of interest?<br><br>(for any reason/indication)	◯ Yes<br>◯ No
Reason for hydroxychloroquine/chloroquine use during the time interval of interest?	◯ Pre-exposure COVID prophylaxis<br>◯ Post-exposure COVID prophylaxis<br>◯ Chronic prescription for an existing medical condition<br>◯ Malaria prophlyaxis<br>◯ Other

RECENT PATIENT CONTACT

Reminder:

If you have had COVID-19, we are interested in the 14 days prior to symptom onset. If you have remained healthy throughout the pandemic, we are interested in the last 14 days. In the 14-day time period of interest, I had healthcare-related contact with:

Patient(s) with laboratory-confirmed COVID-19
◯ Yes
◯ No

Patient(s) under investigation (PUI) for COVID-19

Definition:

A SARS-CoV-2 / COVID test was sent on the patient, and/or The patient is in precautionary COVID-related isolation per local policy
◯ Yes
◯ No

Patient(s) not suspected of having COVID-19
◯ Yes
◯ No

Approximately how many known COVID-19 patients did you care for over the time period of interest?
____

Approximately how many patients under investigation for COVID-19 (PUIs) did you care for over the time period of interest?
____

MEDICAL WORKPLACE SETTING(s)

In the 14-day time period of interest, I worked (or saw patients) in the following areas:

	Yes	No
Intensive care unit (ICU)	○	○
Step-down or intermediate-care unit	○	○
Regular hospital floor unit / ward	○	○
Dedicated COVID intensive care unit (ICU)	○	○
Dedicated non-ICU COVID unit or ward	○	○
Emergency Room / Department / Accident & Emergency	○	○
Out-of-hospital emergency medical care	○	○
Operative or procedure area	○	○
Outpatient clinic, testing area, or other ambulatory setting	○	○
Skilled nursing facility or long-term care facility	○	○
Dialysis unit	○	○
Home health / other care delivered in patient's home	○	○
Rehabilitation facility	○	○
Medical Laboratory area	○	○
Non-patient care area (e.g. radiology reading room)	○	○
Administrative area only	○	○

Did you have any close contact with a healthcare worker colleague at your institution during the 14-day time period of interest who was sick with COVID-19 symptoms or who was later diagnosed with COVID-19?

○ Yes
○ No

Exposures involving LABORATORY-CONFIRMED COVID-19 patients.

In the 14-day time period of interest, I was exposed to the following involving patients with confirmed COVID-19:

	Yes	No
Inside a negative-pressure room with a patient	○	○
Inside a non-negative-pressure room with a patient	○	○
Within 3 feet of a patient	○	○
In the room with a patient continuously for 45 minutes or longer	○	○
In the room with a patient cumulatively for more than 1 hour over the course of a day or shift	○	○
Physical contact with a patient	○	○
Physical contact with a patient's personal items	○	○
Physical contact with respiratory secretions from a patient	○	○
Performed endotracheal intubation (I was the intubating proceduralist)	○	○
Present in the room during endotracheal intubation (but was not the proceduralist)	○	○
Performed endotracheal extubation (I pulled the tube)	○	○
Present in the room during endotracheal extubation (but did not pull the tube myself)	○	○
Performed open suctioning of secretions or body fluid of a patient	○	○
Performed closed suctioning of secretions or body fluid of a patient	○	○
Present in the room during nebulizer use	○	○

Been in the room with a patient on non-invasive positive pressure ventilation (e.g. CPAP, BPAP)	◯	◯
Adjusted the mask of a patient on non-invasive positive pressure ventilation (e.g. CPAP, BPAP)	◯	◯
Been in the room with a patient on high-flow nasal cannula	◯	◯
Placed or adjusted a patient's high flow nasal cannula	◯	◯
Performed bronchoscopy (I was the bronchoscopist)	◯	◯
Present in the room during bronchoscopy (but was not the bronchoscopist)	◯	◯
Performed rigid bronchoscopy (I was the bronchoscopist)	◯	◯
Present in the room during rigid bronchoscopy (but was not the proceduralist)	◯	◯
Performed open tracheotomy (I was the proceduralist)	◯	◯
Present in the room during open tracheotomy (but was not the proceduralist)	◯	◯
Performed percutaneous tracheostomy (I was the proceduralist)	◯	◯
Present in the room during percutaneous tracheostomy (but was not the proceduralist)	◯	◯
Participated in cardiopulmonary resuscitation of a patient who did not have an advanced airway (e.g. endotracheal tube) at some point during my participation	◯	◯
Participated in cardiopulmonary resuscitation of a patient who had an advanced airway in place throughout my participation in the event	◯	◯

Recognized a failure or breach in my personal protective equipment (PPE) during an interaction with a patient or while removing PPE after an interaction	○	○

HOW OFTEN did you experience the following exposures involving a LABORATORY-CONFIRMED COVID-19 patient during the 14-day time period of interest?

Number of times you intubated a known COVID patient in the time interval of interest? ________________________

Number of times you were present for intubation of a known COVID patient (but not performed the intubation yourself) in the time interval of interest? ________________________

Number of times you extubated a known COVID patient in the time interval of interest? ________________________

Number of times you were present during extubation of a known COVID patient (but did not pull the tube yourself)? ________________________

Number of times you performed bronchoscopy in a known COVID patient in the time interval of interest? ________________________

Number of times you were present for bronchoscopy in a known COVID patient (but not beein the bronchoscopist yourself) in the time interval of interest? ________________________

Number of times you performed a tracheotomy in a known COVID patient in the time interval of interest? ________________________

Number of times you were present for bronchoscopy in a known COVID patient (but not been the bronchoscopist yourself) in the time interval of interest? ________________________

Number of times you performed open suctioning of a known COVID patient in the time interval of interest? ________________________

Number of times you manipulated the CPAP or BPAP mask of a known COVID patient in the time interval of interest? ________________________

Number of times you participated in cardiopulmonary resuscitation of involving known COVID patients in the time interval of interest? ________________________

INTUBATION TECHNIQUES

How often were the following techniques used during intubations of LABORATORY-CONFIRMED COVID-19 PATIENTS during the 14-day time period of interest?

	Used in ALL intubations	Used in SOME intubations	Not used at all	I do not know
Intubation in negative pressure room	◯	◯	◯	◯
Rapid sequence intubation, no bag-mask before first attempt	◯	◯	◯	◯
Bag-mask ventilation at some point after drugs pushed	◯	◯	◯	◯
Direct laryngoscopy	◯	◯	◯	◯
Video laryngoscopy	◯	◯	◯	◯
After ETT placement: bag-mask ventilation WITH a viral filter between ETT and ambu bag	◯	◯	◯	◯
After ETT placement: bag-mask ventilation WITHOUT a viral filter between ETT and ambu bag	◯	◯	◯	◯
After ETT placement: direct to ventilator without manual bagging	◯	◯	◯	◯
Intubation box covering patient's head	◯	◯	◯	◯
Tarp, sheet, or other barrier over patient's head during intubation	◯	◯	◯	◯

I used (or was present during use) an intubation technique not listed above which was specifically employed to reduce risk of transmission to healthcare workers

◯ Yes
◯ No

Specify other intubation technique details

____

When caring for LABORATORY-CONFIRMED COVID-19 patients over the time period of interest:

What respiratory protection did you MOST FREQUENTLY utilize in the following circumstances?

During AEROSOL GENERATING PROCEDURES?

Aerosol-generating procedures:

Intubation Extubation Open respiratory suctioning Bronchoscopy Delivery of nebulized medication Induced sputum collection Use of non-invasive positive pressure ventilation Tracheotomy Cardiopulmonary resuscitation

- ◯ No respiratory protection
- ◯ Home-made or improvised mask (cloth, etc)
- ◯ NEW medical/surgical mask
- ◯ RE-USED medical/surgical mask
- ◯ NEW disposable respirator mask (e.g. N95, FFP2, FFP3)
- ◯ RE-USED disposable respirator mask (e.g. N95, FFP2, FFP3)
- ◯ Powered Air Purifying Respirator (PAPR)
- ◯ Re-usable elastomeric respirator
- ◯ Other respirator
- ◯ Other

Specify other respiratory protection: ______________________

During direct patient contact? (when aerosol generating procedures were NOT being performed)

- ◯ No respiratory protection
- ◯ Home-made or improvised mask (cloth, etc)
- ◯ NEW medical/surgical mask
- ◯ RE-USED medical/surgical mask
- ◯ NEW disposable respirator mask (e.g. N95, FFP2, FFP3)
- ◯ RE-USED disposable respirator mask (e.g. N95, FFP2, FFP3)
- ◯ Powered Air Purifying Respirator (PAPR)
- ◯ Re-usable elastomeric respirator
- ◯ Other respirator
- ◯ Other

Specify other respiratory protection: ______________________

When present on the unit housing COVID patients but not in a patient room or in direct contact with a patient?

(i.e. when working on the ward outside a room)

- ◯ No respiratory protection
- ◯ Home-made or improvised mask (cloth, etc)
- ◯ NEW medical/surgical mask
- ◯ RE-USED medical/surgical mask
- ◯ NEW disposable respirator mask (e.g. N95, FFP2, FFP3)
- ◯ RE-USED disposable respirator mask (e.g. N95, FFP2, FFP3)
- ◯ Powered Air Purifying Respirator (PAPR)
- ◯ Re-usable elastomeric respirator
- ◯ Other respirator
- ◯ Other

Specify other respiratory protection: ______________________

During the 14-day period of interest, did you have ANY contact with a known COVID patient while not wearing any respiratory protection? (i.e. no mask)

- ◯ Yes
- ◯ No

During the 14-day period of interest, did you have ANY contact with a known COVID patient while wearing a simple medical/surgical mask or other non-respirator-level respiratory protection?

- ◯ Yes
- ◯ No

During the 14-day period of interest, were you EVER present for an Aerosol-Generating Procedure while wearing a simple medical/surgical mask or other non-respirator-level respiratory protection?

Aerosol-generating procedures:

Intubation Extubation Open respiratory suctioning Bronchoscopy Delivery of nebulized medication Induced sputum collection Use of non-invasive positive pressure ventilation Tracheotomy Cardiopulmonary resuscitation

○ Yes
○ No

Exposures involving "PATIENTS UNDER INVESTIGATION for COVID-19"
(also known as patients with "SUSPECTED COVID-19" or "COVID PUIs")

Patients under investigation include those:
1) In whom a SARS-CoV-2 / COVID-19 test was sent, or
2) Placed in COVID-related precautionary isolation according to local protocol

In the 14-day time period of interest, I was exposed to the following involving patients under investigation for COVID-19:

	Yes	No
Inside a negative-pressure room with a patient	○	○
Inside a non-negative-pressure room with a patient	○	○
Within 3 feet of a patient	○	○
In the room with a patient continuously for 45 minutes or longer	○	○
In the room with a patient cumulatively for more than 1 hour over the course of a day or shift	○	○
Physical contact with a patient	○	○
Physical contact with a patient's personal items	○	○
Physical contact with respiratory secretions from a patient	○	○
Performed endotracheal intubation (I was the intubating proceduralist)	○	○
Present in the room during endotracheal intubation (but was not the proceduralist)	○	○
Performed endotracheal extubation (I pulled the tube)	○	○
Present in the room during endotracheal extubation (but did not pull the tube myself)	○	○
Performed open suctioning of secretions or body fluid of a patient	○	○

Performed closed suctioning of secretions or body fluid of a patient	◯	◯
Present in the room during nebulizer use	◯	◯
Been in the room with a patient on non-invasive positive pressure ventilation (e.g. CPAP, BPAP)	◯	◯
Adjusted the mask of a patient on non-invasive positive pressure ventilation (e.g. CPAP, BPAP)	◯	◯
Been in the room with a patient on high-flow nasal cannula	◯	◯
Placed or adjusted a patient's high flow nasal cannula	◯	◯
Performed bronchoscopy (I was the bronchoscopist)	◯	◯
Present in the room during bronchoscopy (but was not the bronchoscopist)	◯	◯
Performed rigid bronchoscopy (I was the bronchoscopist)	◯	◯
Present in the room during rigid bronchoscopy (but was not the proceduralist)	◯	◯
Performed open tracheotomy (I was the proceduralist)	◯	◯
Present in the room during open tracheotomy (but was not the proceduralist)	◯	◯
Performed percutaneous tracheostomy (I was the proceduralist)	◯	◯
Present in the room during percutaneous tracheostomy (but was not the proceduralist)	◯	◯
Participated in cardiopulmonary resuscitation of a patient who did not have an advanced airway (e.g. endotracheal tube) at some point during my participation	◯	◯

Participated in cardiopulmonary resuscitation of a patient who had an advanced airway in place throughout my participation in the event	○	○
Recognized a failure or breach in my personal protective equipment (PPE) during an interaction with a patient or while removing PPE after an interaction	○	○

When caring for PATIENTS UNDER INVESTIGATION for COVID-19 patients over the time period of interest:

What respiratory protection did you MOST FREQUENTLY utilize in the following circumstances?

During AEROSOL GENERATING PROCEDURES?<br><br>Aerosol-generating procedures include:<br><br>Intubation Extubation Open respiratory suctioning Bronchoscopy Delivery of nebulized medication Induced sputum collection Use of non-invasive positive pressure ventilation Tracheotomy Cardiopulmonary resuscitation	◯ No respiratory protection<br>◯ Home-made or improvised mask (cloth, etc)<br>◯ NEW medical/surgical mask<br>◯ RE-USED medical/surgical mask<br>◯ NEW disposable respirator mask (e.g. N95, FFP2, FFP3)<br>◯ RE-USED disposable respirator mask (e.g. N95, FFP2, FFP3)<br>◯ Powered Air Purifying Respirator (PAPR)<br>◯ Re-usable elastomeric respirator<br>◯ Other respirator<br>◯ Other
Specify other respiratory protection:	__________________________________
During direct patient contact? (when aerosol generating procedures were NOT being performed)	◯ No respiratory protection<br>◯ Home-made or improvised mask (cloth, etc)<br>◯ NEW medical/surgical mask<br>◯ RE-USED medical/surgical mask<br>◯ NEW disposable respirator mask (e.g. N95, FFP2, FFP3)<br>◯ RE-USED disposable respirator mask (e.g. N95, FFP2, FFP3)<br>◯ Powered Air Purifying Respirator (PAPR)<br>◯ Re-usable elastomeric respirator<br>◯ Other respirator<br>◯ Other
Specify other respiratory protection:	__________________________________
When present on the unit housing patients under investigation for COVID but not in a patient room or in direct contact with a patient?<br><br>(i.e. when working on the ward outside a room)	◯ No respiratory protection<br>◯ Home-made or improvised mask (cloth, etc)<br>◯ NEW medical/surgical mask<br>◯ RE-USED medical/surgical mask<br>◯ NEW disposable respirator mask (e.g. N95, FFP2, FFP3)<br>◯ RE-USED disposable respirator mask (e.g. N95, FFP2, FFP3)<br>◯ Powered Air Purifying Respirator (PAPR)<br>◯ Re-usable elastomeric respirator<br>◯ Other respirator<br>◯ Other
Specify other respiratory protection:	__________________________________

Exposures involving non-COVID patients

In the 14-day time period of interest, I was exposed to the following involving non-COVID patients:

Did you perform (or were you present during) AEROSOL GENERATING PROCEDURES involving non-COVID patients in the 14-day time period of interest?

Aerosol-generating procedures include:

Intubation Extubation Open respiratory suctioning Bronchoscopy Delivery of nebulized medication Induced sputum collection Use of non-invasive positive pressure ventilation Tracheotomy Cardiopulmonary resuscitation

◯ Yes
◯ No

How many aerosol-generating procedures involving non-COVID patients were you present for during the 14-day time period of interest?

____

Exposures OUTSIDE THE HEALTHCARE SETTING

In the 14-day time period of interest, did you have any of the following exposures outside the medical setting?

	Yes	No
In-person contact with a person with known COVID-19	○	○
In-person in contact with a person with symptoms of COVID-19 (but not laboratory-confirmed)	○	○
Lived in a household with someone with laboratory-confirmed COVID-19	○	○
Lived in a household with someone with symptoms of COVID-19 (but not laboratory-confirmed)	○	○
Participated in a gathering of more than 10 people	○	○
Dined in a restaurant, been to a bar, or similar (if only taking food/drink away after picking up, answer NO)	○	○
Shopped in a store (e.g. grocery store, or similar)	○	○
Used public transportation	○	○

PERSONAL PROTECTIVE EQUIPMENT (PPE) POLICIES

In the 14-day time period of interest, what local policies or recommendations were in place at your institution?

(if you work at more than one institution, answer these questions regarding the place where you spent the most time during the time period of interest)

Recommended by your institution to enter the room of a known COVID-19 patient (assume no recent aerosol-generating procedures were performed)

- ◯ No respiratory protection
- ◯ Home-made or improvised mask (cloth, etc)
- ◯ NEW medical/surgical mask
- ◯ RE-USED medical/surgical mask
- ◯ NEW disposable respirator mask (e.g. N95, FFP2, FFP3)
- ◯ RE-USED disposable respirator mask (e.g. N95, FFP2, FFP3)
- ◯ Powered Air Purifying Respirator (PAPR)
- ◯ Re-usable elastomeric respirator
- ◯ Other respirator
- ◯ Other

Specify other respiratory protection: __________________________________

Recommended by your institution to enter the room of a known COVID-19 patient in which an aerosol-generating procedure will be performed

Aerosol-generating procedures include:

Intubation Extubation Open respiratory suctioning Bronchoscopy Delivery of nebulized medication Induced sputum collection Use of non-invasive positive pressure ventilation Tracheotomy Cardiopulmonary resuscitation

- ◯ No respiratory protection
- ◯ Home-made or improvised mask (cloth, etc)
- ◯ NEW medical/surgical mask
- ◯ RE-USED medical/surgical mask
- ◯ NEW disposable respirator mask (e.g. N95, FFP2, FFP3)
- ◯ RE-USED disposable respirator mask (e.g. N95, FFP2, FFP3)
- ◯ Powered Air Purifying Respirator (PAPR)
- ◯ Re-usable elastomeric respirator
- ◯ Other respirator
- ◯ Other

Specify other respiratory protection: __________________________________

Does your institution allow "extended use" or "re-use" of disposable N95 or FFP1/FFP2/FFP3 masks?

Extended use = using the same mask, without removing, for more than one patient. Re-use: taking the mask off and putting it back on multiple times

- ◯ Yes
- ◯ No
- ◯ My institution does not have a policy for this
- ◯ I do not know my institution's policy over the time period of interest

My institution employed COVID-only units where personal protective equipment was not doffed between patients during the time period of interest

- ◯ Yes
- ◯ No
- ◯ I do not know

Does your institution utilize PPE observers to monitor proper PPE donning and doffing?

- ◯ Yes, always present and/or available
- ◯ Yes, sometimes present or available
- ◯ No
- ◯ I do not know

What policies were in place at your institution over the time interval in question regarding PPE brought in from home?	◯ Freely allowed to bring in and use PPE from home<br>◯ Allowed but not encouraged to use home PPE<br>◯ Discouraged from using home PPE<br>◯ Home PPE explicitly not allowed<br>◯ My institution does not have a policy on this<br>◯ I do not know my institutions policy over the time period in question
Did you use any PPE brought in from home in the last two weeks (or two weeks prior to onset of COVID symptoms)?<br><br>Check all that apply	☐ I did not use any PPE from home during the time period of interest<br>☐ Respirator-level respiratory protection (N95, FFP2 or FFP3, reusable elastomeric respirator, other similar)<br>☐ Simple respiratory protection (surgical or medical mask, cloth/fabric mask)<br>☐ Barrier protection (re-purposed or improvised gown/gloves/head covering)
Have you received in-person PPE donning and doffing training at your current institution?	◯ Yes<br>◯ No
Comments (optional)<br><br>Please do not leave any personally identifiable information, regarding yourself or another!	______________________________

Instructions to complete this survey: 1. The submit button below will submit your data. 2. It is imperitive that we compare your exposures with others at your institution who have NOT become ill. 3. Please personally forward this survey to five individuals at your institution. This will maximize the impact of your responses and experiences and help us determine what exposures are placing us at risk. The survey link will be displayed after you click "submit."

Click "submit" below to submit your data. Thank you! Please forward this survey to any healthcare worker colleagues who have developed COVID-19.

The survey link will be displayed after you click submit.
