# Supplementary material for: Assessing coronavirus disease 2019 (COVID-19) transmission to healthcare personnel: The global ACT-HCP case-control study
Source: Infect Control Hosp Epidemiol. 2020 Sep 9:1–7. doi: 10.1017/ice.2020.455 (PMC7542323; doi:10.1017/ice.2020.455)
Supplement: Supplementary file 1 [file icesup.zip › S0899823X20004559sup003.pdf]

<u>The ACT-HCW study (Assessing COVID-19 Transmission to HealthCare Workers)</u>

A Public Health Project of the COVIDBRONCH Initiative

Have you, or a healthcare worker you know, been diagnosed with COVID-19?

Please contribute to the ACT-HCW Study by telling us about workplace exposures which might have contributed to your illness.

<u>Only takes 15 minutes</u>: https://redcap.link/act-hcw

<u>WHO</u>: Any healthcare worker who has been ill with (or tested positive for) COVID-19. Healthcare workers healthy throughout the pandemic who have worked in the last two weeks can also complete the survey as a healthy control.

<u>WHY</u>: Urgent need to identify medical workplace exposures potentially associated with an increased risk of transmission of COVID-19 to healthcare workers.

<u>WHAT</u>: Online survey regarding workplace exposures (patient contact, PPE use, PPE policies at your institution).

<u>WHEN</u>: Now!

<u>WHERE</u>: https://redcap.link/act-hcw (easy to complete on your phone!)

<u>Note</u>: This research has been approved as Exempt Research upon IRB review at Vanderbilt University Medical Center, Nashville, TN, USA (IRB #200677, approval date 4/13/2020). Meets CFR 46.104 (d) category (2) exemption criteria, on the basis of: 1) less than minimal risk to participants; 2) research includes survey-only interactions, and 3) no identifying data recorded.

CONTACT US:

henricolt@gmail.com, robert.j.lentz@vumc.org, or fabien.maldonado@vumc.org
